# Needs and health-related quality of life domains relevant to people in Europe with advanced cancer in need of palliative care: a systematic review of qualitative research

**DOI:** 10.1007/s11136-025-04129-0

**Published:** 2026-02-18

**Authors:** Catalina Lizano-Barrantes, Clara Amat-Fernandez, Olatz Garin, Ricardo Luer-Aguila, Yolanda Pardo, Leslye Rojas-Concha, Melissa S.Y. Thong, Giovanni Apolone, Cinzia Brunelli, Augusto Caraceni, Norbert Couespel, Nanne Bos, Mogens Groenvold, Stein Kaasa, Gennaro Ciliberto, Claudio Lombardo, Ricardo Pietrobon, Gabriella Pravettoni, Aude Sirven, Hugo Vachon, Alexandra Gilbert, Galina Velikova, Montse Ferrer, EUonQoL Working Group, Tudor Ciuleanu, Milana Mitrić, Ivica Ratosa, Michal Chovanec, Maria Vieito, Héctor Aguilar, Eva Ruiz, Karin Ahlberg, Eda Tanrikulu Simsek, Mahmut Gumus, Inke van Braak, Caitriona Higgins, Laura Pinnavaia, Carina Dantas, Tapani Kalmi

**Affiliations:** 1 Health Services Research Group, Hospital del Mar Research Institute, Barcelona, Spain; 2 Department of Pharmaceutical Care and Clinical Pharmacy, Faculty of Pharmacy, Universidad de Costa Rica, San Jose, Costa Rica; 3 CIBER en Epidemiología y Salud Pública, CIBERESP, ISCIII, Madrid, Spain; 4 Department of Medicine and Life Sciences, Universitat Pompeu Fabra, Barcelona, Spain; 5 Department of Psychiatry and Legal Medicine, Universitat Autònoma de Barcelona, Barcelona, Spain; 6 Palliative Care Research Unit, Department of Geriatrics and Palliative Medicine GP, Copenhagen University Hospital – Bispebjerg, Copenhagen, Denmark; 7 Unit of Cancer Survivorship, German Cancer Research Center (DKFZ), Heidelberg, Germany; 8 Direzione Scientifica, Fondazione IRCCS Istituto Nazionale Dei Tumori, Milan, Italy; 9 Organization of European Cancer Institutes, Brussels, Belgium; 10 Dipartimento di Scienze Cliniche e di Comunità, Dipartimento di Eccellenza 2023-2027, Università Degli Studi Di Milano, Milan, Italy; 11 European Cancer Organisation (ECO), Brussels, Belgium; 12 Netherlands Institute for Health Services Research (Nivel), Utrecht, The Netherlands; 13 Department of Public Health and Bispebjerg / Frederiksberg Hospital, University of Copenhagen, Copenhagen, Denmark; 14 Oslo Universitetssykehus HF, Oslo, Norway; 15 IRCCS National Cancer Institute “Regina Elena” (on behalf of Digital institute for cancer outcomes research (DIGICORE), Brussels, Belgium), Rome, Italy; 16 SporeData OÜ, Tallinn, Estonia; 17 Istituto Europeo Di Oncologia IRCCS, Milan, Italy; 18 Unicancer, Paris, France; 19 European Organisation for Research and Treatment of Cancer, Brussels, Belgium; 20 Leeds Institute of Medical Research at St James’s, University of Leeds, Leeds, UK; 21 Leeds Teaching Hospitals NHS Trust, Leeds, UK

**Keywords:** Quality of life, Neoplasms, Cancer, Palliative care, Systematic review, Qualitative research

## Abstract

**Purpose:**

There is a need for a comprehensive summary of qualitative research on the health-related quality of life (HRQoL) of people with advanced cancer requiring palliative care. We aim to systematically review qualitative studies on outcomes, needs, experiences, preferences, concerns and HRQoL of people in Europe with advanced cancer requiring palliative care over the last decade.

**Methods:**

Protocol registered (www.crd.york.ac.uk/PROSPERO, CRD42024575065). The search was conducted in PubMed and Scopus, from 2013 onward. Inclusion criteria: qualitative studies addressing constructs related to the HRQoL of adults with cancer requiring palliative care in Europe. Abstracts and full texts were reviewed, data extracted, and risk of bias assessed independently by two researchers. A thematic analysis stratified by study objective was performed, grouping the emerging themes into categories (primary outcome).

**Results:**

Of 18,256 articles identified, 20 fulfilled the inclusion criteria: 10 studies with a generic objective (whole palliative process or end-of-life phase), and 10 with specific focuses. Five categories (35 themes) emerged from the studies with generic focuses: ‘Psychological Function’ (*n* = 15), ‘Clinical Management’ (*n* = 8), ‘Symptoms and Physical Function’ (*n* = 6), ‘Social Function’ (*n* = 5), and ‘End-of-life’ (*n* = 1). Themes from the 7 studies focusing on treatment, services, and self-management also fitted into these categories, adding ‘Spiritual Well-being’.

**Conclusion:**

These findings emphasise the predominance of the psychological function domain in cancer patients requiring palliative care, including cancer-related anxiety and distress, coping mechanisms, control and decision-making, and fearing and expecting death. Additionally, clinical management unmet needs were identified in health care, information and communication, and end-of-life settings (home vs. hospital).

**Supplementary Information:**

The online version contains supplementary material available at 10.1007/s11136-025-04129-0.

## Plain language abstract

Differences across Europe in access to palliative care can affect the symptoms suffered by patients with advanced cancer. Many questionnaires measuring quality of life among oncology patients in palliative care failed to address the whole range of their concerns. Through a systematic review of the literature, we identified 20 studies where these patients express their needs, experiences, preferences, and the impact on their quality of life. Beyond the traditional physical dimension, our results highlight the predominance of the psychological and spiritual dimensions among people in Europe with advanced cancer requiring palliative care over the last decade. Also, these patients often comment the importance of clinical management, which usually is not included in quality of life instruments, to consider the way the healthcare professionals address and inform them of each step, and to support shared decision-making, including where to spend their end-of-life stage: at home or in a hospital. New questionnaires to measure correctly the many dimensions identified by patients with advanced cancer will allow the healthcare systems in European countries improve their understanding and allow for policy changes to better support them at this last stage of their lives.

**Supplementary Information:**

The online version contains supplementary material available at 10.1007/s11136-025-04129-0.

## Introduction

In 2022, the global cancer landscape estimated approximately 20 million new cases and 10 million deaths, with Europe accounting for a fifth of these cases (22.4%) and deaths (20.4%), despite having less than 10% of the world’s population (9.6%) [1]. Advances in cancer diagnosis, treatment, and surveillance have extended the lives of patients with advanced cancer that is unlikely to be cured or controlled [2], leading to an increased demand for palliative care [3]. High-quality palliative care remains accessible to only 14% of the world’s population, primarily in European countries [4], but with marked geographic disparities among them in life expectancy, maximum lifespan [5, 6], and services [7].

Disparities across Europe in access to palliative care can affect the symptoms patients suffer [8]. These differences highlight the urgent need for equitable palliative care solutions that comprehensively address the entire spectrum of patients’ needs, particularly in domains often overlooked [9, 10]. According to a systematic meta review of 40 reviews encompassing studies conducted mainly in Europe, North America, and China [11], advanced cancer significantly impairs health-related quality of life (HRQoL), with patients facing severe challenges and unmet needs that vary due to cultural and healthcare contexts [9, 10]. Consequently, improving the HRQoL of this population has become a central focus of Europe’s Beating Cancer Plan for 2021–2023 [12] and Mission Cancer [13].

Monitoring symptoms and HRQoL is crucial in advanced cancer care, as it enhances healthcare professionals´ awareness, helping to better anticipate patients’ changing needs [14] and improving outcomes [15]. Addressing unmet needs early in the palliative care process is essential, as they can amplify the trauma of living with a life-limiting illness and impact HRQoL [16]. Nevertheless, the abovementioned systematic meta review [11] pointed out that HRQoL in people receiving palliative care remains understudied, with many trials treating this as a secondary outcome. Furthermore, despite the increase in specialized palliative care services across Europe in the last decade [7], routine monitoring of HRQoL by patient-reported outcome measures (PROM) remains underutilized in this setting [17].

A systematic review [18] evaluating the impact of specialized palliative care on oncology patients’ HRQoL included 12 studies from countries with advanced healthcare systems, and highlighted significant gaps in addressing social and spiritual dimensions comprehensively. Another systematic review on HRQoL instruments for patients in advanced cancer [19] revealed that only 12 out of the 39 identified were specific for people receiving palliative care in general, terminal phase, or nearing the end of life, and noted that many of these tools failed to address the full spectrum of needs in oncological palliative care, not covering all relevant HRQoL dimensions. Nine of these instruments were developed in Canada and the United States, and only three in Europe (Sweden and the United Kingdom) [19].

Qualitative research provides insights into these nuanced and subjective experiences, identifying needs and informing the development of more effective, patient-centred assessment tools. However, there is no comprehensive summary of qualitative research that identifies the HRQoL issues specifically relevant for people with advanced cancer nowadays. Therefore, our study aims to systematically review qualitative studies focusing on disease-related outcomes, needs, experiences, preferences, concerns and quality of life of people in Europe with advanced cancer in need of palliative care over the last decade.

This review was conducted as part of the European project EUonQoL [20], which aims to develop a new patient-reported outcome measures toolkit for assessing HRQoL among patients with cancer across Europe. The synthesis of the evidence from this review has been useful for the development of the EUonQoL toolkit, and could be of interest for other HRQoL instruments for people with advanced cancer in need of, or receiving, palliative care, specially to identify those domains usually unmet in the traditional HRQoL conceptual models.

## Methods

The reporting of this systematic review adheres to the guidelines outlined in the Preferred Reporting Items for Systematic Reviews and Meta-Analyses (PRISMA) [21], and the protocol is registered (CRD42024575065 in www.crd.york.ac.uk/PROSPERO).

### Eligibility criteria

The inclusion criteria considered for this review were: studies employing qualitative methods, including mixed-method approaches, that examined disease-related outcomes, needs, preferences, concerns, worries, or quality of life, in persons with advanced cancer in need of palliative care defined as [22]: projected prognosis < 12 months with Eastern Cooperative Oncology Group (ECOG) performance status ≥ 2 [23], or referred to a specialist palliative care team for receiving non-curative treatment purely for symptom control. This study focused exclusively on peer-reviewed articles published in European languages, with samples from the 27 European Union (EU) countries, the United Kingdom (UK), and the 16 associated countries (Supplementary Table 1 lists all 44 countries).

Exclusion criteria were studies on children, on adolescents or young adults (AYAs); individuals undergoing active cancer treatment or surviving cancer; patients with multimorbidity; partners, caregivers, or healthcare professionals; studies aiming to explore dimensions specific to tumour location; specific populations, such as minorities; and studies collecting data exclusively before 2013, to focus on people receiving palliative care in the last decade to capture the present situation in this setting. AYAs (cancer diagnosis at the upper age limit of 39 years [24]) were excluded, since their specific needs were out of the scope of the EUonQoL project, which focuses on adults.

### Information sources

The search was initially conducted in MEDLINE via PubMed on March 6th, 2023, and subsequently updated on July 8th, 2024, including both Pubmed and Scopus databases. Since PubMed covers up to 92% of references in Cochrane reviews [25], pairing it with Scopus guarantees enough coverage.

### Search strategy

The search strategy in PubMed and Scopus had 4 sections (Supplementary Table 2): one focused on the type of population (survivors, patients under treatment, or palliative patients), a second one on the pathology (neoplasm), a third section regarding the constructs of interest (related to quality of life), and a last one referring to relevant issues such as preferences and concerns. Due to differences in their filtering capabilities, the search strategy in PubMed included both MeSH and text word terms, while in Scopus it included a country-based filter. Several search strategies were tested, and the final choice was based on the comparison between a search restricted to MeSH subheadings versus broader terms in PubMed. The latter strategy was selected because it yielded 16% more articles.

While the initial search included all patient groups within the cancer continuum as part of the EUonQoL project, the results presented here focus specifically on those in need of palliative care. This decision arises from their significantly different experiences to patients undergoing active treatment or surviving cancer [26].

### Selection process

The screening process was performed using Covidence™ software (www.covidence.org): each title and abstract was reviewed independently by two out of six researchers (CAF, OG, MF, CLB, RL, LRC), following a pilot test to establish standardized criteria; and disagreements were resolved through discussions involving third-party reviewers.

### Data collection process

Each study underwent full text review and data extraction, also conducted independently by two researchers (CAF, RL, OG, YP, RB, CLB, MT, LRC), using an ad-hoc data extraction form. A third reviewer verified the data extraction tables for accuracy and completeness.

### Data items

The information extracted included: study characteristics (aim, design, country, data collection, recruitment methodology, theoretical approach, and qualitative method); sample characteristics (tumour location, sample size, age, sex, setting, and time since palliative care); and reporting of information (use of guidelines for qualitative research, themes, subthemes and verbatims).

### Study risk of bias

To evaluate the risk of bias in the included studies, we employed the Specialist Unit for Review Evidence Qualitative Studies Critical Appraisal (SURE) checklist [27], which consists of 10 items rated as either ‘Yes’, ‘Can’t tell’, or ‘No’. Researchers who conducted the data extraction also performed this assessment. Studies that received a ‘No’ rating on three or more of the ten items were classified as ‘poor quality’.

### Outcomes

The primary outcome was established as the themes and subthemes (and specific verbatims, when necessary) extracted from the included studies. To mitigate researchers’ interpretation bias, the themes and subthemes were extracted literally from each article.

### Synthesis methods

The EUonQoL project followed the most widely applied theoretical model for HRQoL [28], the Wilson & Cleary´s framework [29]. Consequently, this HRQoL model served as the basis for the thematic analysis, which was conducted by a panel of researchers in two stages with the software Microsoft Whiteboard. The first stage employed a deductive approach to distribute themes and subthemes into categories following the Wilson and Cleary framework, with discussion among the researchers of the panel until consensus was reached. In the second stage, an inductive approach was used to identify subcategories emerging from the data, following an iterative process until achieving consensus.

### Reporting bias assessment

A sensitivity analysis was planned with only studies deemed of good quality (less than three of the ten items of the SURE checklist with a negative qualification). Additionally, to prevent the overrepresentation in the synthesis of results from studies focusing on particular constructs, the thematic analysis was stratified based on the objective of the qualitative study, to consider exclusively those with a general focus for the primary analysis. Specific-focus studies were considered only in a secondary separate analysis. Therefore, the primary thematic analysis broadly addressed disease-related outcomes, needs, experiences, preferences, concerns, and quality of life.

## Results

### Study selection

**Fig. 1:**
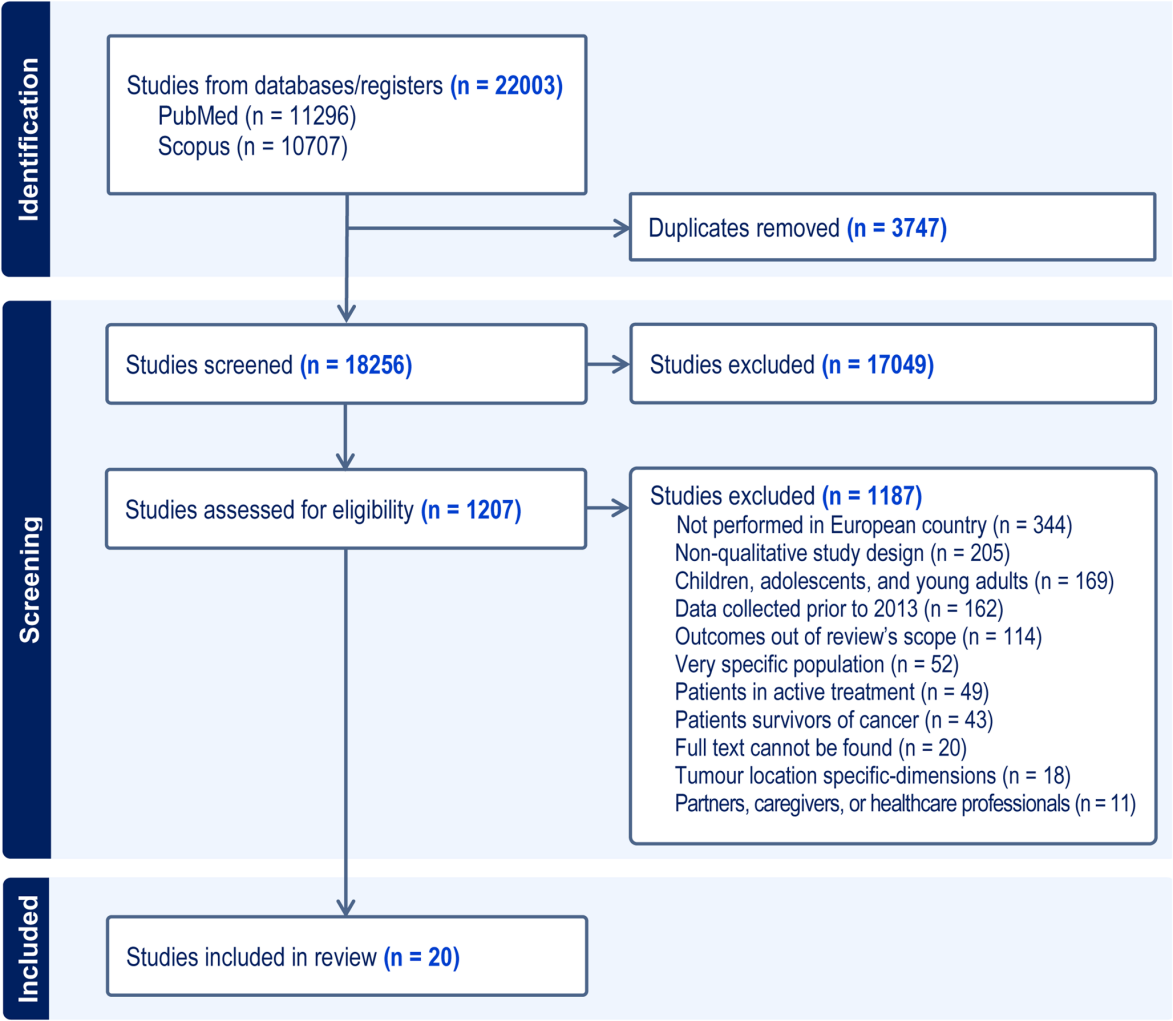
Selection process overview – PRISMA flow-chart

A total of 18,256 articles were identified across PubMed and Scopus. The study selection process is detailed in the PRISMA flowchart (Fig. 1). Following title and abstract screening, 1207 manuscripts underwent full-text review. The most frequent reasons for exclusion of articles at this stage were not set in a European country (31%), non-qualitative study design (18%), focus on children, adolescents and young adults (15%), and data collected before 2013 (14%). Finally, 20 qualitative studies on palliative care met the inclusion criteria and proceeded to the data extraction phase.

### Study characteristics

The characteristics of the included studies are summarized in Table 1. The countries with the highest number of studies were Denmark (*n* = 5), Germany (*n* = 3), and Sweden (*n* = 3). The data collection methods used most often were semistructured interviews guided by predefined questions, but allowing open responses (*n* = 13), and in-depth interviews with flexible, detailed explorations of individual experiences (*n* = 4). Ten studies included patients with various tumour locations, and women represented around 50% of the sample in most studies on the non-gender related cancer.

**Table 1:** Characteristics of the included studies.

	Number of studies
Total	20
Country​	
Denmark	5
Germany	3
Sweden	3
United Kingdom	2
Norway	2
Spain	2
Turkey	1
Italy	1
Israel	1
Year of publication	
2015-2016	4
2017-2018	6
2019-2020	4
2021-2022	3
2023-2024	3
Qualitative approach​	
Semi-structured interviews	13
In-depth interviews	4
Cognitive debriefings	1
Narrative interviews and supplementary participating observation	1
Open interviews and diaries	1
Tumour location​	
Multiple locations	10
Oesophageal and Stomach	3
Not reported	3
Lung	2
Breast	1
Colorectal	1
Gender (% of women)*	
<25%	7
25-49%	5
50-74%	6
≥75	1
Setting	
Outpatient (home, clinic or hospital)	10
Inpatient (hospital)	3
Hospice or Palliative cancer care service (home)	3
Inpatient & Hospice (home)	1
Inpatient & Outpatient	2
Not reported	1
Focus	
General experiences, preferences, needs, concerns in:	
Whole palliative process	6
End-of-life phase	4
Specific objectives on:	
Treatment, services, self-management	7
Pain experiences	2
Spiritual well-being	1

*Excluding gender-dependent tumours

Half of the studies were conducted with outpatient participants, while 30% involved those in hospital or hospice settings. Ten studies (50%) aimed to generically explore the experiences, needs, concerns, and preferences of people with cancer throughout the whole palliative process [30–35] or at the end-of-life phase [36–39]. The remaining ten studies focused on specific objectives, such as treatment, services, or self-management [40–46], pain experiences [47, 48], and spiritual well-being [49].

### Risk of bias in studies

Supplementary Table 3 shows the quality of the studies included, assessed following the SURE checklist [27]. All the studies addressed at least nine of the ten items of the SURE checklist, except for two that met eight items [36, 46]. Due to these good SURE results, none of the studies were assessed as poor quality, hence the sensitivity analysis was not needed.

### Results of individual studies

Table 2 shows the characteristics of the studies and the identified themes. The six qualitative studies with a generic focus addressing the whole palliative process (all but one [31]) were based on phenomenology and used semi-structured interviews, with sample sizes ranging from 6 to 17 participants, each identifying 1 to 4 themes (14 in total). Two of the studies with a generic focus addressing the end-of-life phase were based on phenomenology and two on Grounded Theory, and all but one [37] used semi-structured interviews. The sample size of these studies ranged from 3 to 20, identifying 12 themes in total.

**Table 2:** Characteristics and results of included studies categorized by their objectives

Study (Author, Year, Country, setting)	Methods (Qualitative approach, data collection)	Participants (Sample size, age, % women, time since PT, M, Di, and De, tumour location)	Aim of study	Themes identified (as reported in the study)
A. GENERIC FOCUS ON DISEASE-RELATED OUTCOMES, EXPERIENCES, PREFERENCES, NEEDS, CONCERNS, AND QoL				
A1. WHOLE PALLIATIVE PROCESS				
Dalhammar 2023 [30] Sweden outpatient (hospital)	Phenomenology Semi-structured interviews	*n* = 12 55–87 years 17% women Time not reported Stomach and oesophageal	To explore how patients newly diagnosed with incurable oesophageal and gastric cancer manage everyday life	• Striving towards normality in an unpredictable situation
Hofheinz 2016 [31] Germany Outpatient	Does not specify In-depth interviews	*n* = 6 42–85 years 22% women Time not reported Stomach and oesophageal	To assess patient preferences for a new hypothetical palliative chemotherapy of gastric cancer in Germany, using a Choice-based conjoint analysis approach, in patients with previous or ongoing chemotherapy exposure	• Ability to self-care • Treatment tolerability • Survival benefit
Laursen 2019 [32] Denmark Inpatient & outpatient	Phenomenology with hermeneutic interpretation Semi-structured interviews	*n* = 17 54–74 years 41% women 1–23 months since Di Oesophageal	To illuminate the ways in which incurable oesophageal cancer disrupts the patients’ lives and how the patients experience and adapt to life with the disease in order to suggest palliative care interventions	• Eating difficulties forces the patients to withdrawal from social interactions • Loss of control and confidence in own body caused by the symptoms and treatment • Clinging to life by keeping things as normal as possible and focus on the present • The challenge of managing one’s own illness when continuity is lacking
Loughran 2019 [33] UK Outpatient (home and hospital)	Phenomenology Semi-structured interviews	*n* = 6 58–77 years 50% women Time not reported Multiple locations	To address this paucity of information by recording and describing the lived experiences of people living with incurable cancer, the effects on their lives, their views on rehabilitation, and their perceived rehabilitation needs in palliative care setting	• Functional difficulties experienced by people living with incurable cancer. • Rehabilitation needs in a palliative setting.
Madsen 2019 [34] Denmark Palliative cancer care service (home)	Phenomenology with hermeneutic interpretation Semi-structured interviews	*n* = 10 50–86 years 50% women Time not reported Multiple locations	To explore patients’ experiences of transitions during the course of incurable cancer	• Everyday life changes. • Approaching end of life.
Rodríguez-Prat 2022 [35] Spain Outpatient (clinic and hospital)	Phenomenology Semi-structured interviews	*n* = 8 29–70 years 75% women Time not reported Multiple locations	To explore how patients with advanced cancer understand control, in terms of underlying beliefs, attitudes, and expectations consistent with self-efficacy, in different dimensions of their life, their illness, and their healthcare	• Factors that influence the perception of control. • Perceiving control over an uncontrollable illness.
A.2. END-OF-LIFE PHASE				
Dobrina 2016 [36] Italy Hospice (home)	Phenomenology Semi-structured interviews	*n* = 11 48–75 years 45% women 5–7 days before De Multiple locations	To explore needs and wishes in the last week of life of patients	• Remaining attached to my life • Detach myself from life, immediately • Dealing with the dying process • Starting to think of life without me
IvzoriErel 2022 [37] Israel Inpatient & Hospice (home)	Phenomenology In-depth interviews	*n* = 20 31–77 years 55% women ≤ 6 months before De Location not reported	To explore the experience of a sense of place among individuals at the end-of-life receiving care at home via home-hospice or in a hospital	• Body as a place • Sense of place towards the place of care • The lack of a sense of place
López-Salas 2024 [38] Spain Not reported	Grounded theory semi-structured interviews	*n* = 3 2/3 < 65 years 0% women ≤ 6 months before De Location not reported	To obtain more in-depth insight into unmet needs, primarily in patients with end-stage cancer nearing death	• Physical well-being. • Emotional well-being • Social well-being: adapted living space and mobility and economic resources. • Information and autonomy in decision-making
Stanze 2019 [39] Germany Inpatient & outpatient	Grounded theory semi-structured interviews	*n* = 17 45–78 years 18% women Time not reported Lung	To understand the needs, explore the experiences and meaning of living with advanced cancer at the end of life, and develop strategies for improved patient-centred care in Germany	• Redefining one’s own existence
B. SPECIFIC OBJECTIVES				
B.1. EXPERIENCES WITH TREATMENT, SERVICES AND SETTING				
Aumann 2016 [40] Germany Outpatient (clinic)	Do not specify Semi-structured interviews	*n* = 18 48–76 years 39% women Time not reported Lung	To ascertain a range of experiences of patients with lung cancer and to make recommendations regarding the improvement of treatment based on their preferences	• Experiences and preferences during the treatment day • Experiences with physicians • Experiences with health insurance • Treatment-related experiences and preferences of the patients that influence psychosocial factors
Bergqvist 2017 [41] Sweden Outpatient (hospital)	Phenomenology cognitive debriefings	*n* = 20 40–80 years 100% women 1–35 years since Di, 1–15 years since M Breast	To investigate breast cancer patients’ motives, perceptions, and experiences of late lines of palliative oncologic treatment	• The decision process • Personal motives and goals • The treatment itself
Håkanson 2015 [42] Sweden Inpatient (hospital)	Phenomenology Narrative interviews and supplementary participating observation	*n* = 9 57–76 years 56% women 1 day-16 weeks before De Multiple locations	To enhance the depth of existing knowledge about meanings and experiential outcomes of bodily care in the context of an inpatient specialist palliative setting	• Maintaining and losing body capability • Breaching borders of bodily integrity. • Being comforted and relieved in bodily care situations • Being left in distress with unmet needs
Maersk 2019 [43] Denmark Outpatient (home)	Grounded theory In-depth interviews	*n* = 22 38–88 years 64% women Time not reported Multiple locations	To explore how the identity of people with advanced cancer is influenced by their experiences of living at home	• Managing the home to enable activities • Maintaining the privacy of home • Displaying and hiding symbols of identity
Nysæter 2022 [44] Norway outpatient (home)	Grounded Theory Semi-structured interviews	*n* = 9 47–90 years 22% women 1–6 months since PT Location not reported	To explore the preferences for home care over time among adult patients with cancer in the late palliative phase to enable home death.	• Hope and trust to get the care I need to die at home
Peoples 2017 [45] Denmark Outpatient (home)	Phenomenology Semi-structured interviews	*n* = 73 68.3 years (mean) 47% women Time not reported Multiple locations	To describe and explore how people with advanced cancer manage occupations in their everyday lives.	• Conditions influencing occupations in everyday life. • Self-developed strategies to manage occupations
Sonderup Tarp 2024 [46] Denmark Inpatient (hospital)	Do not specify Semi-structured interviews	*n* = 10 52–90 years 50% women < 1–4 years since Di Multiple locations	To explore the experienced assessment-process and treatment of palliative symptoms, as well as the experienced symptom burden, in patients with metastatic upper GI cancer.	• The treatment of physical symptoms. • Existential, social, and psychological symptoms • The assessment of symptoms.
B.2. PAIN EXPERIENCES				
Dunham 2017 [47] UK Hospice	Phenomenology open interview/ Diaries	*n* = 9 67–88 years 22% women Time not reported Multiple locations	To consider how the older person constructs the experience of cancer pain and how this is informed by expectations and experiences	• Better to be old than to be dying with cancer • Maintaining control and independence • Loss of identity-adapting and grieving for a former self • Dislike of analgesia. • Denial of pain
Erol 2018 [48] Turkey inpatient (hospital)	Phenomenology Semi-structured interviews	*n* = 16 62.75 years (mean) 19% women 3 months ± 4.5 since Di Multiple locations	To explore the pain experiences of patients with advanced cancer and how they manage with pain, and to present a view of pain management approaches of nurses from the perspectives of the patients	• Pain perception and patient experiences • Effects of pain on daily life • Pain management and management strategies • Patients’ perspectives about nurses’ approaches to pain
B.3. SPIRITUAL WELL-BEING				
Rohde 2017 [49] Norway Outpatient (hospital)	Hermeneutic In-depth interviews	*n* = 20 34–75 years 40% women Time not reported Colorectal	To explore Spiritual Well-Being in colorectal cancer patients in the palliative phase undergoing chemotherapy	• Relationships with self and others • Existential issues • Specifically religious and/or spiritual beliefs and practices

*PT* palliative treatment, *M* metastases; *Di* primary diagnostic, *De* death , *QoL* quality of life

The ten studies with a specific focus drew on a wider range of qualitative designs, including Grounded Theory, cognitive debriefings, or narrative methods, and generally involved larger samples [40–49]. The number of themes emerging from these studies was 39: 25 themes from the seven studies exploring experiences on treatment, services, and self-management [40–46], 11 themes from the two studies on pain [47, 48], and 3 themes from the one on spiritual well-being [49].

Although several studies combined different settings, those primarily conducted in outpatient or home contexts often emphasized themes related to daily life, occupations, and continuity of care, regardless whether they had general [30–39] or specific objectives [40–49]. Again whichever the focus of the study, those including either inpatient or hospice participants would highlight more frequently bodily experiences and existential concerns [36, 37, 39, 42, 46]. The 3 studies centred on oesophageal and gastric cancers often focused on disruptions related to eating, social interaction, and treatment tolerability [30–32], while those involving multiple tumour sites produced broader psychosocial and existential themes [33–35, 37, 42–46, 48].

### Results of synthesis: primary thematic analysis of studies with generic objectives

**Fig. 2:**
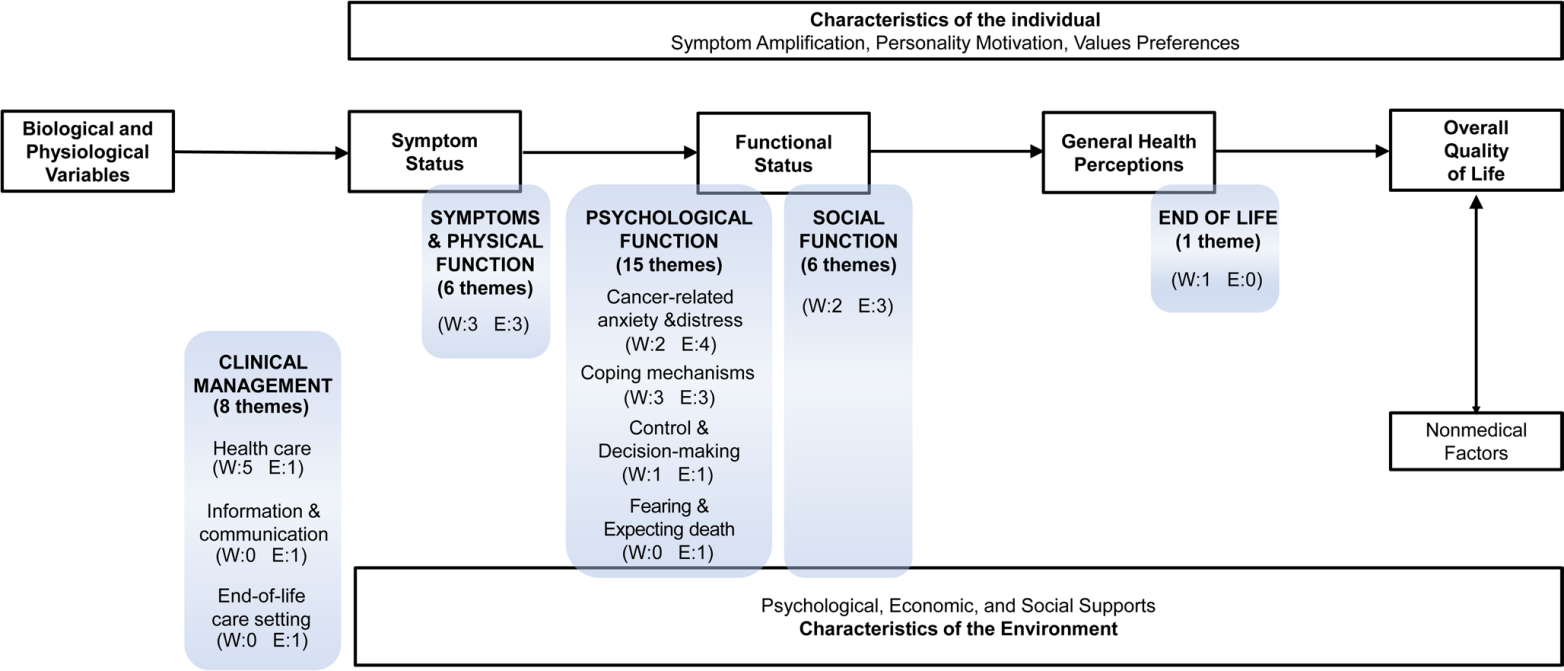
Overview of the results of the primary thematic analysis of qualitative studies with a generic focus within the Wilson and Cleary HRQoL framework [29]. Clear boxes show the framework developed by Wilson and Cleary [29].Shadowed, rounded boxes show the categories and subcategories that emerged from the thematic analysis (number of themes/subthemes identified in the primary studies)

As represented in clear boxes in Fig. 2, the framework developed by Wilson and Cleary [29] conceptualizes HRQoL as a multidimensional construct comprising five components: biological and physiological variables, symptom status, functional status, general health perceptions, and overall quality of life, alongside the individual and environmental characteristics that influence these components. Rounded shadowed boxes illustrate the categories and subcategories derived from the thematic analysis of themes and subthemes identified in the 10 studies with a generic focus, and they are positioned within the original components of the framework, except for the category of Clinical Management which lies outside the framework domains. Three categories (‘Symptoms & Physical Function’, ‘Psychological Function’, and ‘Social Function’) primarily align with the ‘Functional Status’ component, and the category of ‘End of Life’ is situated within the ‘General Health Perceptions’ component. Table 3 presents the list of all themes and subthemes (or descriptions and verbatims when subthemes were not reported), aggregated into categories and subcategories from the studies with a generic focus. Certain themes were placed into multiple categories or subcategories, based on the content of the subthemes or verbatims.

**Table 3:** Themes and subthemes (or verbatims) distributed into categories

A. *PSYCHOLOGICAL FUNCTION (15 THEMES)*	
A.1. *Cancer-related anxiety and distress (6 themes)*	
^W^*Everyday life changes [34]	Normal life changes
^W^*Factors that influence the perception of control [35]	Uncertainty about future suffering; Character traits underlying the need for control
^E^ Starting to think of life without me [36]	“Unshared Worries”: Although the family still encouraged patients that “things will be better soon,” patients reported that they were aware of what was happening and preferred not to talk with them about the end of life.
^E^*The lack of a sense of place [37]	A lack of sense of place, focused on detachment from physical and social environments and loss of sense of place
^E^*Emotional well-being [38]	Tracking emotional well-being; Addressing emotional distress; Self-identity and self-esteem
^E^*Redefining one’s own existence [39]	Powerlessness
A.2. *Coping mechanisms (6 themes)*	
^W^ Striving towards normality in an unpredictable situation [30]	Trying to comprehend the disease: the origin of the disease; the nature of the disease Dealing with the consequences of illness: handling symptoms and problems; maintaining a strong body and spirit; finding ways to engage with family and friends Re-evaluating what is important in everyday life: reflecting on what is important in life; downshifting
^W^ Clinging to life by keeping things as normal as possible and focus on the present [32]	“One day at a time”: To maintain usual daily routines and living day to day are a way of clinging to life and, therefore, not relinquishing what they know and who they are
^W^*Perceiving control over an uncontrollable illness [35]	Adjusting the focus of control
^E^ Remaining attached to my life [36]	“I know my illness is getting serious, but through my great will, I will get better… I only need to get some strength back and get this pain under control, so I can get up and do all the nice things I used to do, and start an “almost” normal life again”
^E^*Emotional well-being [38]	Seeking out distractions.
^E^*Redefining one’s own existence [39]	Rearrangement of everyday life; Individual way of dealing consciously with the treatment; Design of the therapeutic setting; Learning to live with the threat; Keeping one’s composure
A.3. *Control and decision making (2 themes)*	
^W^*Perceiving control over an uncontrollable illness [35]	Perceived control over subjective wellbeing: Relationship between maintaining control over the situation, being able to make choices about their lives, about treatments and to promote personal wellbeing.
^W^*Information and autonomy in decision-making [38]	Patient´s wills and preferences: Needs related to informed decision-making are associated with a willingness to ensure patient inclusion and autonomy in decisions that are made throughout the end of-life period.
A.4. *Fearing and expecting death (1 theme)*	
^E^ Dealing with the dying process [36]	“Waiting in fear”
B. *CLINICAL MANAGEMENT (8 THEMES)*	
B.1. *Health care (6 themes)*	
^W^ Treatment tolerability [31]	Patients with previous CT experience consider a survival benefit accompanied by high quality of life, i.e. being able to self-care and receiving a treatment with good tolerability, as more important than an additional survival benefit per se
^W^ Survival benefit [31]	
^W^ The challenge of managing one’s own illness when continuity is lacking [32]	Some of the issues that were described by the patients when continuity was lacking include: difficulty getting to appointments and navigating the system; health care professionals who do not understand their situation; and lack of support and symptom management
^W^ Rehabilitation needs in a palliative setting [33]	Participants considered rehabilitation as a beneficial and very important part of their cancer care, in some cases more important than medical treatment
^W^*Factors that influence the perception of control [35]	Sense of lack of care as a source of loss of control
^E^ Social well-being: adapted living space and mobility and economic resources [38]	Cover costs of technical and/or orthotic and prosthetic devices; Cover costs of professional care; Personal space; Domestic mobility; Transfers by vehicle
B.2.* Information & communication (1 theme)*	
^E^*Information and autonomy in decision-making [38]	Reliable and transparent information
B.3.* End-of-life care setting (1 theme)*	
^E^ Sense of place towards the place of care [37]	The sense of home versus the hospital, focused on the sense of place towards home and hospital
C. *SYMPTOMS AND PHYSICAL FUNCTION (6 THEMES)*	
^W^ Ability to self-care [31]	Patients evaluate their palliative chemotherapy based on the extent of survival benefit associated with a high perceived quality of life, which they predominantly characterized as being able to self-care and receiving a treatment with good tolerability
^W^ Loss of control and confidence in own body caused by the symptoms and treatment [32]	“Being in a zombie-like state”: patients being hit by activity loss and being homeless in their own body; it describes the interruption of everyday life caused by pain, exhaustion and illness
^W^ Functional difficulties experienced by people living with incurable cancer [33]	Participants reported several functional difficulties related to their cancer or its treatment. Physical symptoms such as pain, fatigue, and reduced mobility were the most common symptoms experienced and had the greatest impact
^E^ Detach myself from life, immediately [36]	“I know I will die soon, but I was hoping not to suffer so much… I wish all this could be over soon!”
^E^ Body as a place [37]	The sick body and the body as a place, focused on the experience of estrangement with and disappointment from the body
^E^ Physical well-being [38]	Symptom monitoring and control; Sleep & Rest; Cleanliness and personal hygiene; Nutrition; Physical activity
D. *SOCIAL FUNCTION (5 THEMES)*	
^W^ Eating difficulties forces the patients to withdrawal from social interactions [32]	“Sense of isolation”: Some patients feel embarrassed and undignified when eating in the company of others, and they often end up withdrawing from social situations
^W^*Everyday life changes [34]	People changing behaviour; Changes hurting loved ones
^E^*Emotional well-being [38]	Affective relationships; Social Relationships
^E^*Redefining one’s own existence [39]	The entire illness experience is codetermined contextually by the role shift in the social fabric, which begins at the time of diagnosis
^E^*The lack of a sense of place [37]	A lack of sense of place, focused on detachment from physical and social environments and loss of sense of place
E. *END OF LIFE (1 THEME)*	
^W^ Approaching end of life [34]	Approaching death; Preparing for leaving; Holding on to life; Connecting with places and belongings

^W^ Studies addressing the whole palliative process;

^E^ Studies addressing the end-of-life phase;

*Themes categorized into more than one category or subcategory according to the content of the subthemes

#### A. Psychological function

This category contained the most themes (15 out of 35), with ‘Cancer-related anxiety and distress’ and ‘Coping mechanisms’ being the most predominant subcategories.

A.1. ‘Cancer-related anxiety and distress’: six themes considered the complex emotional landscape of the end-of-life phase, including confronting mortality, experiencing detachment from their environment, addressing distress, adapting to everyday life changes, grappling with uncertainty about future suffering, or redefining their existence in the face of powerlessness [34–39].

A.2. ‘Coping mechanisms’: six themes centred around patients pursuing normality in the face of an unpredictable illness, balancing efforts to comprehend the disease, manage its consequences, maintain emotional well-being, or redefine their existence by adjusting priorities, seeking control, and focusing on the present [30, 32, 35, 36, 38, 39].

A.3. ‘Control and Decision-making’: the two themes in this subcategory emphasize the importance of perceiving control over their illness and well-being, valuing the ability to make informed decisions about their lives and treatments, which promotes autonomy and aligns with their personal preferences and end-of-life needs [35, 38].

A.4. ‘Fearing and expecting death’: a single theme from a study focusing on the end-of-life phase described these dual feelings, closely connected to the perception of waiting without doing anything, which was experienced as particularly difficult [36].

#### B. Clinical management

B.1. ‘Health care’: most themes in this subcategory emerged from studies of the whole palliative process and considered aspects such as treatment, continuity, specific care needs, and costs [31–33, 35, 38]. A study involving patients with previous chemotherapy experience highlighted that receiving a treatment with good tolerability was considered more important than achieving additional survival benefits [31].

B.2. ‘Information & Communication’: The sole theme in this subcategory emerged from one study focusing on the end-of-life phase, highlighting the need for reliable and transparent information. The interviewees emphasized that they often felt as if healthcare professionals were not honest and forthright when sharing information with them [38].

B.3. ‘End-of-life care setting’: this subcategory also included only one theme from a study focusing on the end-of-life phase, reflecting the conflicting and ambivalent feelings towards home and the hospital [37].

#### C. Symptoms / Physical function

This category included 6 themes, three from the whole palliative process studies [31–33] and three from end-of-life phase ones [36–38]. ‘Physical well-being’ was often associated with the ability to self-care, maintain symptom control, and receive tolerable treatment, though some patients expressed feelings of estrangement from their bodies or a desire to detach from life to escape suffering.

#### D. Social function

The five themes within this category emphasized the negative effects on family dynamics, social networks, and participation in social activities [32, 34, 37–39].

#### E. End-of-life

The end-of-life category has a single theme that emerged from a study focusing on the whole palliative process [34]. Patients reflected on death being a general living condition for all people, but with an increased awareness on ending life and dying which prompted four sub-themes: approaching death, preparing for leaving, holding on to life, and connecting with places and belongings.

### Results of synthesis: secondary thematic analysis of studies with specific objectives

The results of the thematic analysis from the 10 studies with specific objectives are shown in the Supplementary Tables. From the 7 qualitative studies focusing on treatment, services and self-management (Supplementary Table 4) [40–46], most of the 25 themes identified fit in the same categories as the primary thematic analysis, except for a new category entitled ‘Spiritual’. The 11 themes from studies exploring pain (Supplementary Table 5) [47, 48] pointed out the importance of ‘Psychological Function’ and ‘Symptoms & Physical Function’, with very few themes in the ‘Clinical Management’, and no themes in the rest of categories. The 3 themes emerging from the study centred on spiritual well-being (Supplementary Table 6) [49] highlighted spiritual and social function. Finally, no themes emerged within the ‘End-of-life’ category in any of the studies with specific objectives.

## Discussion

The review of qualitative studies on outcomes, needs, experiences, preferences, concerns, and quality of life for adults in Europe with advanced cancer in need of palliative care identified 20 studies meeting inclusion criteria from the 18,256 articles screened. The primary thematic analysis of 35 themes and subthemes from the 10 studies with a generic focus revealed that most of them aligned with Wilson and Cleary’s Functional Status domain (‘Symptoms and Physical Function’, ‘Psychological Function’, and ‘Social Function’ categories), while only one theme about ‘End-of-life’ was classified in the General Health Perceptions domain. Notably, the ‘Clinical Management’ category emerged as a significant concern outside the framework domains. Additionally, in the 10 studies with specific objectives, the most frequently explored construct was the experience with treatment, services, and self-management, addressed by 7 studies.

### Psychological function

The primary thematic analysis revealed the relevant impact of advanced cancer on patients’ ‘Psychological Function’ in studies involving people in need of palliative care. This was also observed in the secondary analysis of studies with objectives focused on pain and on treatment, services, and self-management. The ‘Cancer-related anxiety and distress’ subcategory was the most frequent across studies both with generic and specific focuses. Our results align with two systematic reviews of unmet care needs in advanced cancer patients [9, 10], which identified the psychological domain as one of the three most commonly reported, with emotional support being the most frequently unmet need within this domain. These findings are further supported by another systematic review of qualitative research on life-limiting illnesses [50], which highlights the emotional state as a critical aspect of quality of life from patients’ perspectives. Additionally, a systematic review of population-based quality indicators for end-of-life cancer care [51] emphasized the lack of validated quality indicators for psychological needs.

The themes in both our analyses also underscore the importance of patients perceiving control over their illness and well-being, valuing informed decision-making to maintain autonomy and align with their personal preferences and end-of-life needs. This is consistent with findings from a systematic review focused on palliative care of adults with cancer [52], which emphasizes the role of advanced cancer care planning in offering patients an opportunity to consider and document their preferences for the last phase of their life. Ensuring that patients are given this chance early and repeatedly could help address their emotional needs more effectively [52, 53].

### Symptoms and physical function

The ‘Symptoms and Physical Function’ impact of advanced cancer in people in need of palliative care appears to be transversal across generic studies focusing on the whole palliative process and the end-of-life phase, and studies with specific aims (on pain and on treatment, services, and self-management), generally related with the ability to self-care and maintain symptom control. Our results are consistent with systematic reviews on unmet care needs and quality of life in people with advanced cancer, highlighting the physical domain [9, 10], including being able to do the things you used to, and symptoms such as fatigue and pain.

### Social function

The impact in ‘Social Function’ was less pronounced than in other domains in the primary thematic analysis of studies with a generic focus. Nonetheless, related themes also emerged from studies focused on treatment, services, self-management, and spiritual well-being in the secondary thematic analysis. Another systematic review also identified social unmet needs among people with advanced cancer, though they were not within the most prominently reported [9]. Nevertheless, the importance of socializing as part of quality of life is supported by qualitative research, which highlights it as a significant aspect for individuals with life-limiting illnesses receiving palliative care [50].

### General health perceptions

Within the Wilson and Cleary domain of ‘General Health Perceptions’, the emergence in the primary thematic analysis of a category titled ‘End-of-life’ is expected, given the characteristics of our target population. Although this category includes only one theme, its rise in a generic study addressing the whole palliative process [34] supports the relevance of this aspect, highlighting how patients transition into a state of living where their awareness of death becomes dominant. Furthermore, the four qualitative studies specifically centred on the end-of-life phase [36–39] also confirm the importance of this domain from the perspective of stakeholders other than patients. The critical role of preparation for death in quality of life is emphasized in several systematic reviews, which identify it as a key patient priority [50, 54], and highlights the importance of creating opportunities for end-of-life discussions [52, 54–56].

In the secondary thematic analysis of studies with specific aims a new ‘Spiritual’ category appears. Notably, though spirituality was mentioned in the results of 6 generic studies, this content was not elevated to a theme or subtheme by the authors of the original articles, suggesting that this topic was not prioritized in their thematic analyses. The spiritual domain was identified as important for quality of life in a systematic review of qualitative studies in patients with life-limiting illnesses receiving palliation [50]. Nonetheless, it was not found as one of the prominently unmet needs among people with advanced cancer in another systematic review, mainly including quantitative studies [9]. These apparently contradictory findings may be explained by the lack of a spiritual domain within most of the HRQoL tools [10, 19]. In the same line, the underrepresentation of this aspect was also highlighted in a systematic review of population-based quality indicators for end-of-life cancer care [51].

### Clinical management

The ‘Clinical Management’ category emerged prominently in our systematic review, being the second most prevalent category of themes in the primary thematic analysis among studies with a generic objective. In addition, there are seven studies focusing specifically on experiences with treatment, services, and self-management which also support the importance of this aspect. Our findings align with systematic reviews highlighting some deficits on the healthcare provision and information [9, 10, 51, 52] among the most common complaints, alongside financial concerns [10]. Two prominent issues were: being informed about treatment benefits and side effects [9], and effective communication skills for patient-physician discussions regarding profound changes in disease trajectory [52]. Furthermore, the important gaps in validated quality indicators for structure, processes of care, and communication, reinforce the need for robust indicators to improve clinical management and quality of life [51]. These insights underscore the central role of clinical management in addressing patients’ evolving needs [57] and enhancing their quality of life in palliative care.

### Strengths and limitations

The results of this review should be interpreted with caution. First, half of the included studies had specific objectives and did not aim to comprehensively identify needs and QoL issues relevant to people with advanced cancer in need of palliative care. To address this, the primary thematic analysis included exclusively findings from studies with a generic focus to avoid overrepresentation of results from those with specific objectives. Second, the studies meeting the inclusion criteria were carried out in only 5 EU-27 countries (Denmark, Germany, Norway, Spain, Italy), 3 associated countries (Sweden, Turkey, Israel), and the UK, and the few European countries that are not part of the Horizon Europe program were excluded from the search. Therefore, people from the Eastern and Central European regions were underrepresented. Third, an inherent limitation of the studies included in this review is that not being fluent in the country’s language was an exclusion criterion for half of them (11 out of 20). Thus, migrants and other minority populations are underrepresented in the results. Furthermore, many of the included studies provide limited information on demographic and clinical characteristics of their participants; for example, education level was reported only in 5 articles, ECOG scores in 3, and living situation in 2.

Lastly, despite the challenges associated with conducting studies in palliative medicine, the studies found addressed different phases of the palliative process and tumour locations. Our initial search was broad to maximize sensitivity, which explains why almost a thousand of the studies included in the full-text review were later found to meet the exclusion criteria (country, methods, population, dates, or outcomes). Additionally, good-quality evidence was identified, since all the included studies had less than three out of the ten items of the SURE checklist with a negative qualification, and 3 fulfilled positively the whole SURE checklist. The least reported information in the studies was the ‘relationship between the researcher and the participant’ (present only in 30% of studies), even though it is considered in previous checklists for qualitative studies [58, 59]. Considering the good methodological quality of the included studies and their variety of target populations across the whole palliative process, the findings from our review could be valuable to know which concerns are relevant to people with advanced cancer in need of palliative care. They could also be of value to support clinical practice, for instance by guiding communication training, informing care pathways, and strengthening multidisciplinary support.

## Conclusions

Findings from this systematic review show the predominance of the psychological function domain among people with advanced cancer in need of palliative care, including cancer-related anxiety and distress, coping mechanisms, control and decision-making, and fearing and expecting death. Beyond the traditional physical and social function domains, our results further highlight the need of a clinical management domain in HRQoL instruments to address health care, information and communication, and home vs. hospital settings for the end of life; this is usually not included, considering it covered by patient-reported experience measures. Finally, the results also support the importance of addressing often overlooked transcendent domains, such as end-of-life and spiritual well-being. These findings provide valuable guidance for the development of HRQoL instruments, including the EUonQoL toolkit, to better reflect the diverse and multidimensional needs of patients. Ensuring to encompass these domains is essential to support shared decision-making, enhance care delivery, and inform equitable healthcare policies. At the same time, the limited number of identified studies highlights the need for further qualitative research in this population, especially in the Eastern and Central regions of Europe, to broaden the evidence base and strengthen future PROM development.

## Electronic Supplementary Material

Below is the link to the electronic supplementary material.

Supplementary information
