# Correction: Needs and health-related quality of life domains relevant to people in Europe with advanced cancer in need of palliative care: a systematic review of qualitative research

**DOI:** 10.1007/s11136-026-04268-y

**Published:** 2026-07-07

**Authors:** Catalina Lizano-Barrantes, Clara Amat-Fernandez, Olatz Garin, Ricardo Luer-Aguila, Yolanda Pardo, Leslye Rojas-Concha, Melissa S. Y. Thong, Giovanni Apolone, Cinzia Brunelli, Augusto Caraceni, Norbert Couespel, Nanne Bos, Mogens Groenvold, Stein Kaasa, Gennaro Ciliberto, Claudio Lombardo, Ricardo Pietrobon, Gabriella Pravettoni, Aude Sirven, Hugo Vachon, Alexandra Gilbert, Galina Velikova, Montse Ferrer, EUonQoL Working Group, Tudor Ciuleanu, Ivica Ratosa, Michal Chovanec, Maria Vieito, Héctor Aguilar, Eva Ruiz, Karin Ahlberg, Eda Tanrikulu Simsek, Mahmut Gumus, Inke van Braak, Caitriona Higgins, Laura Pinnavaia, Carina Dantas, Tapani Kalmi

**Affiliations:** 1 Health Services Research Group, Hospital del Mar Research Institute, Barcelona, Spain; 2 Department of Pharmaceutical Care and Clinical Pharmacy, Faculty of Pharmacy, Universidad de Costa Rica, San Jose, Costa Rica; 3 CIBER en Epidemiología y Salud Pública, CIBERESP, ISCIII, Madrid, Spain; 4 Department of Medicine and Life Sciences, Universitat Pompeu Fabra, Barcelona, Spain; 5 Unicancer, Paris, France; 6 Department of Psychiatry and Legal Medicine, Universitat Autònoma de Barcelona, Barcelona, Spain; 7 Palliative Care Research Unit, Department of Geriatrics and Palliative Medicine GP, Copenhagen University Hospital – Bispebjerg, Copenhagen, Denmark; 8 Unit of Cancer Survivorship, German Cancer Research Center (DKFZ), Heidelberg, Germany; 9 Direzione Scientifica, Fondazione IRCCS Istituto Nazionale Dei Tumori, Milan, Italy; 10 Organization of European Cancer Institutes, Brussels, Belgium; 11 Dipartimento Di Scienze Cliniche E Di Comunità, Università Degli Studi Di Milano, Dipartimento Di Eccellenza, 2023-2027 Milan, Italy; 12 European Cancer Organisation (ECO), Brussels, Belgium; 13 Netherlands Institute for Health Services Research (Nivel), Utrecht, The Netherlands; 14 Department of Public Health and Bispebjerg / Frederiksberg Hospital, University of Copenhagen, Copenhagen, Denmark; 15 Oslo Universitetssykehus HF, Oslo, Norway; 16 IRCCS National Cancer Institute “Regina Elena” (On Behalf of Digital Institute for Cancer Outcomes Research (DIGICORE), Brussels, Belgium), Rome, Italy; 17 SporeData OÜ, Tallinn, Estonia; 18 Istituto Europeo Di Oncologia IRCCS, Milan, Italy; 19 European Organisation for Research and Treatment of Cancer, Brussels, Belgium; 20 Leeds Institute of Medical Research at St James’s, University of Leeds, Leeds, UK; 21 Leeds Teaching Hospitals NHS Trust, Leeds, UK

**Correction to: Quality of Life Research (2025) 35:74** **https://doi.org/10.1007/s11136-025-04129-0**

In this article, Tables 1 and 2 were formatted incorrectly. And the title of Table 3 was published as XXX but should have been "Themes and subthemes (or verbatims) distributed into categories". The correct Tables 1 and 2 are provided in this correction.

**Table 1:** Characteristics of the included studies.

	Number of studies
Total	20
Country​	
Denmark	5
Germany	3
Sweden	3
United Kingdom	2
Norway	2
Spain	2
Turkey	1
Italy	1
Israel	1
Year of publication	
2015-2016	4
2017-2018	6
2019-2020	4
2021-2022	3
2023-2024	3
Qualitative approach​	
Semi-structured interviews	13
In-depth interviews	4
Cognitive debriefings	1
Narrative interviews and supplementary participating observation	1
Open interviews and diaries	1
Tumour location​	
Multiple locations	10
Oesophageal and Stomach	3
Not reported	3
Lung	2
Breast	1
Colorectal	1
Gender (% of women)*	
<25%	7
25-49%	5
50-74%	6
≥75	1
Setting	
Outpatient (home, clinic or hospital)	10
Inpatient (hospital)	3
Hospice or Palliative cancer care service (home)	3
Inpatient & Hospice (home)	1
Inpatient & Outpatient	2
Not reported	1
Focus	
General experiences, preferences, needs, concerns in:	
Whole palliative process	6
End-of-life phase	4
Specific objectives on:	
Treatment, services, self-management	7
Pain experiences	2
Spiritual well-being	1

*Excluding gender-dependent tumours

**Table 2:** Characteristics and results of included studies categorized by their objectives

**Study** (Author, Year, Country, setting)	**Methods** (Qualitative approach, data collection)	**Participants** (Sample size, age, % women, time since PT, M, Di, and De, tumour location)	**Aim of study**	**Themes identified** (as reported in the study)
A. GENERIC FOCUS ON DISEASE-RELATED OUTCOMES, EXPERIENCES, PREFERENCES, NEEDS, CONCERNS, AND QoL				
A1. WHOLE PALLIATIVE PROCESS				
**Dalhammar 2023** [30] Sweden outpatient (hospital)	Phenomenology Semi-structured interviews	*n*?=?12 55?87 years 17% women Time not reported Stomach and oesophageal	To explore how patients newly diagnosed with incurable oesophageal and gastric cancer manage everyday life	? Striving towards normality in an unpredictable situation
**Hofheinz 2016** [31] Germany Outpatient	Does not specify In-depth interviews	*n*?=?6 42?85 years 22% women Time not reported Stomach and oesophageal	To assess patient preferences for a new hypothetical palliative chemotherapy of gastric cancer in Germany, using a Choice-based conjoint analysis approach, in patients with previous or ongoing chemotherapy exposure	? Ability to self-care ? Treatment tolerability ? Survival benefit
**Laursen 2019** [32] Denmark Inpatient & outpatient	Phenomenology with hermeneutic interpretation Semi-structured interviews	*n*?=?17 54?74 years 41% women 1?23 months since Di Oesophageal	To illuminate the ways in which incurable oesophageal cancer disrupts the patients? lives and how the patients experience and adapt to life with the disease in order to suggest palliative care interventions	? Eating difficulties forces the patients to withdrawal from social interactions ? Loss of control and confidence in own body caused by the symptoms and treatment ? Clinging to life by keeping things as normal as possible and focus on the present ? The challenge of managing one?s own illness when continuity is lacking
**Loughran 2019** [33] UK Outpatient (home and hospital)	Phenomenology Semi-structured interviews	*n*?=?6 58?77 years 50% women Time not reported Multiple locations	To address this paucity of information by recording and describing the lived experiences of people living with incurable cancer, the effects on their lives, their views on rehabilitation, and their perceived rehabilitation needs in palliative care setting	? Functional difficulties experienced by people living with incurable cancer. ? Rehabilitation needs in a palliative setting.
**Madsen 2019** [34] Denmark Palliative cancer care service (home)	Phenomenology with hermeneutic interpretation Semi-structured interviews	*n*?=?10 50?86 years 50% women Time not reported Multiple locations	To explore patients? experiences of transitions during the course of incurable cancer	? Everyday life changes. ? Approaching end of life.
**Rodríguez-Prat 2022** [35] Spain Outpatient (clinic and hospital)	Phenomenology Semi-structured interviews	*n*?=?8 29?70 years 75% women Time not reported Multiple locations	To explore how patients with advanced cancer understand control, in terms of underlying beliefs, attitudes, and expectations consistent with self-efficacy, in different dimensions of their life, their illness, and their healthcare	? Factors that influence the perception of control. ? Perceiving control over an uncontrollable illness.

A.2. END-OF-LIFE PHASE				
**Dobrina 2016** [36] Italy Hospice (home)	Phenomenology Semi-structured interviews	*n*?=?11 48?75 years 45% women 5?7 days before De Multiple locations	To explore needs and wishes in the last week of life of patients	? Remaining attached to my life ? Detach myself from life, immediately ? Dealing with the dying process ? Starting to think of life without me
**IvzoriErel 2022** [37] Israel Inpatient & Hospice (home)	Phenomenology In-depth interviews	*n*?=?20 31?77 years 55% women ??6 months before De Location not reported	To explore the experience of a sense of place among individuals at the end-of-life receiving care at home via home-hospice or in a hospital	? Body as a place ? Sense of place towards the place of care ? The lack of a sense of place
**López-Salas 2024** [38] Spain Not reported	Grounded theory semi-structured interviews	*n*?=?3 2/3?<?65 years 0% women ??6 months before De Location not reported	To obtain more in-depth insight into unmet needs, primarily in patients with end-stage cancer nearing death	? Physical well-being. ? Emotional well-being ? Social well-being: adapted living space and mobility and economic resources. ? Information and autonomy in decision-making
**Stanze 2019** [39] Germany Inpatient & outpatient	Grounded theory semi-structured interviews	*n*?=?17 45?78 years 18% women Time not reported Lung	To understand the needs, explore the experiences and meaning of living with advanced cancer at the end of life, and develop strategies for improved patient-centred care in Germany	? Redefining one?s own existence

B. SPECIFIC OBJECTIVES				
B.1. EXPERIENCES WITH TREATMENT, SERVICES AND SETTING				
**Aumann 2016** [40] Germany Outpatient (clinic)	Do not specify Semi-structured interviews	*n*?=?18 48?76 years 39% women Time not reported Lung	To ascertain a range of experiences of patients with lung cancer and to make recommendations regarding the improvement of treatment based on their preferences	? Experiences and preferences during the treatment day ? Experiences with physicians ? Experiences with health insurance ? Treatment-related experiences and preferences of the patients that influence psychosocial factors
**Bergqvist 2017** [41] Sweden Outpatient (hospital)	Phenomenology cognitive debriefings	*n*?=?20 40?80 years 100% women 1?35 years since Di, 1?15 years since M Breast	To investigate breast cancer patients? motives, perceptions, and experiences of late lines of palliative oncologic treatment	? The decision process ? Personal motives and goals ? The treatment itself
**Håkanson 2015** [42] Sweden Inpatient (hospital)	Phenomenology Narrative interviews and supplementary participating observation	*n*?=?9 57?76 years 56% women 1 day-16 weeks before De Multiple locations	To enhance the depth of existing knowledge about meanings and experiential outcomes of bodily care in the context of an inpatient specialist palliative setting	? Maintaining and losing body capability ? Breaching borders of bodily integrity. ? Being comforted and relieved in bodily care situations ? Being left in distress with unmet needs
**Maersk 2019** [43] Denmark Outpatient (home)	Grounded theory In-depth interviews	*n*?=?22 38?88 years 64% women Time not reported Multiple locations	To explore how the identity of people with advanced cancer is influenced by their experiences of living at home	? Managing the home to enable activities ? Maintaining the privacy of home ? Displaying and hiding symbols of identity
**Nysæter 202**2 [44] Norway outpatient (home)	Grounded Theory Semi-structured interviews	*n*?=?9 47?90 years 22% women 1?6 months since PT Location not reported	To explore the preferences for home care over time among adult patients with cancer in the late palliative phase to enable home death.	? Hope and trust to get the care I need to die at home
**Peoples 2017** [45] Denmark Outpatient (home)	Phenomenology Semi-structured interviews	*n*?=?73 68.3 years (mean) 47% women Time not reported Multiple locations	To describe and explore how people with advanced cancer manage occupations in their everyday lives.	? Conditions influencing occupations in everyday life. ? Self-developed strategies to manage occupations
**Sonderup Tarp 2024** [46] Denmark Inpatient (hospital)	Do not specify Semi-structured interviews	*n*?=?10 52?90 years 50% women <?1?4 years since Di Multiple locations	To explore the experienced assessment-process and treatment of palliative symptoms, as well as the experienced symptom burden, in patients with metastatic upper GI cancer.	? The treatment of physical symptoms. ? Existential, social, and psychological symptoms ? The assessment of symptoms.

B.2. PAIN EXPERIENCES				
**Dunham 2017** [47] UK Hospice	Phenomenology open interview/ Diaries	*n*?=?9 67?88 years 22% women Time not reported Multiple locations	To consider how the older person constructs the experience of cancer pain and how this is informed by expectations and experiences	? Better to be old than to be dying with cancer ? Maintaining control and independence ? Loss of identity-adapting and grieving for a former self ? Dislike of analgesia. ? Denial of pain
**Erol 2018** [48] Turkey inpatient (hospital)	Phenomenology Semi-structured interviews	*n*?=?16 62.75 years (mean) 19% women 3 months?±?4.5 since Di Multiple locations	To explore the pain experiences of patients with advanced cancer and how they manage with pain, and to present a view of pain management approaches of nurses from the perspectives of the patients	? Pain perception and patient experiences ? Effects of pain on daily life ? Pain management and management strategies ? Patients? perspectives about nurses? approaches to pain

B.3. SPIRITUAL WELL-BEING				
**Rohde 2017** [49] Norway Outpatient (hospital)	Hermeneutic In-depth interviews	*n*?=?20 34?75 years 40% women Time not reported Colorectal	To explore Spiritual Well-Being in colorectal cancer patients in the palliative phase undergoing chemotherapy	? Relationships with self and others ? Existential issues ? Specifically religious and/or spiritual beliefs and practices

*PT* palliative treatment, *M* metastases; *Di* primary diagnostic, *De* death , *QoL* quality of life

The original article has been corrected.

