# Supplementary material for: Needs and health-related quality of life domains relevant to people in Europe with advanced cancer in need of palliative care: a systematic review of qualitative research
Source: Qual Life Res. 2026 Feb 18;35(3):74. doi: 10.1007/s11136-025-04129-0 (PMC12916547; doi:10.1007/s11136-025-04129-0)
Supplement: Supplementary file 1 — Supplementary information [file 11136_2025_4129_MOESM1_ESM.pdf]

SUPPLEMENTARY INFORMATION

Needs and health-related quality of life domains relevant to people in Europe with advanced cancer in need of palliative care: A systematic review of qualitative research. *Quality of Life Research.* *Catalina Lizano-Barrantes, *Clara Amat-Fernandez, Olatz Garin, Ricardo Luer-Aguila, Yolanda Pardo, Leslye Rojas-Concha, Melissa Thong, Giovanni Apolone, Cinzia Brunelli, Augusto Caraceni, Norbert Couespel, Nanne Bos, Mogens Groenvold, Stein Kaasa, Gennaro Ciliberto, Claudio Lombardo, Ricardo Pietrobon, Gabriella Pravettoni, Aude Sirven, Hugo Vachon, Alexandra Gilbert, Galina Velikova, Montse Ferrer, and the EUonQoL Working Group.

Corresponding authors: Montse Ferrer mferrer@researchmar.net & Olatz Garin ogarin@researchmar.net. Health Service Research Group. Hospital del Mar Research Institute, Barcelona, Spain.

Supplementary table 1 shows the list of European and associated countries in the EUonQoL project.

Supplementary table 2 shows the search strategy conducted in PubMed and Scopus. It included both MeSH and text word terms, and was stratified in 4 sections.

Supplementary table 3 shows the quality of the included studies, assessed following the SURE checklist.

Supplementary table 4 shows the results of thematic analysis of studies focused on treatment, services and self-management.

Supplementary table 5 shows the results of thematic analysis of studies focused on pain.

Supplementary table 6 shows the results of thematic analysis of studies focused on spiritual well-being.

Supplementary checklist 1 shows the PRISMA checklist completed for this systematic review.

Supplementary table 1. List of European Union and associated countries in the EUonQoL project.

**European Union and associated countries**		
Albania	Germany	North-Macedonia
Armenia	Greece	Norway
Austria	Hungary	Poland
Belgium	Iceland	Portugal
Bosnia and Herzegovina	Ireland	Romania
Bulgaria	Israel	Serbia
Croatia	Italy	Slovenia
Cyprus	Kosovo	Slovakia
Czechia	Latvia	Spain
Denmark	Lithuania	Sweden
Estonia	Luxembourg	Tunisia
Faroe Islands	Malta	Turkey
Finland	Moldavia	Ukraine
France	Montenegro	United Kingdom
Georgia	Netherlands	

Supplementary table 2. Search Strategy used for the literature review in PubMed and Scopus.

**PubMed**
("Patient*"[Text Word] OR "Survivor*"[Text Word] OR "Palliative Care"[Text Word] OR "Patient*"[Mesh] OR "Survivor*"[Mesh] OR "Palliative Care"[Mesh])
AND ("Neoplasms"[Mesh] OR "post-cancer" [Title/Abstract] OR "postcancer" [Title/Abstract])
AND ("Quality of Life"[Mesh] OR "perceived health"[Text Word] OR "health status"[Text Word] OR "well-being" [Text Word] OR "wellbeing"[Text Word] OR "Patient Reported Outcome Measures"[Mesh] OR “health-related quality of life”[Text Word] OR “health related quality of life”[Text Word] OR “patient-reported outcome*” [Text Word] OR “patient reported outcome*” [Text Word])
AND (“relevan*”[Text Word] OR “import*”[Text Word] OR “preferences”[Text Word] OR “feelings”[Text Word] OR “needs”[Text Word] OR “issues”[Text Word] OR “concerns”[Text Word] OR “worries”[Text Word] OR “difficulties”[Text Word] OR “limitations”[Text Word] OR “experiences”[Text Word] OR “problems”[Text Word])
FILTERS: Publication Date since 2013

**Scopus**
(TITLE-ABS-KEY ("Patient*" OR "Survivor*" OR "Palliative Care"))
AND (TITLE-ABS-KEY ("Neoplasms" OR "post-cancer" OR "postcancer"))
AND (TITLE-ABS-KEY ("Quality of Life" OR "perceived health" OR "health status" OR "well-being" OR "wellbeing" OR "Patient Reported Outcome Measures" OR "health-related quality of life" OR "patient reported outcome*" OR "health related quality of life" OR "patient-reported outcome"))
AND (TITLE-ABS-KEY ("relevan*" OR "import*" OR "preferences" OR "feelings" OR "needs" OR "issues" OR "concerns" OR "worries" OR "difficulties" OR "limitations" OR "experiences" OR "problems"))
AND PUBYEAR > 2012
AND (EXCLUDE (AFFILCOUNTRY , "United States") OR EXCLUDE (AFFILCOUNTRY , "China") OR EXCLUDE (AFFILCOUNTRY , "Canada") OR EXCLUDE (AFFILCOUNTRY , "Australia") OR EXCLUDE (AFFILCOUNTRY , "Japan") OR EXCLUDE (AFFILCOUNTRY , "South Korea") OR EXCLUDE (AFFILCOUNTRY , "Switzerland") OR EXCLUDE (AFFILCOUNTRY , "Brazil") OR EXCLUDE (AFFILCOUNTRY , "India") OR EXCLUDE (AFFILCOUNTRY , "Taiwan") OR EXCLUDE (AFFILCOUNTRY , "Iran") OR EXCLUDE (AFFILCOUNTRY , "Hong Kong") OR EXCLUDE (AFFILCOUNTRY , "Singapore") OR EXCLUDE (AFFILCOUNTRY , "Russian Federation") OR EXCLUDE (AFFILCOUNTRY , "Mexico") OR EXCLUDE (AFFILCOUNTRY , "New Zealand") OR EXCLUDE (AFFILCOUNTRY , "Malaysia") OR EXCLUDE (AFFILCOUNTRY , "Saudi Arabia") OR EXCLUDE (AFFILCOUNTRY , "Egypt") OR EXCLUDE (AFFILCOUNTRY , "South Africa") OR EXCLUDE (AFFILCOUNTRY , "Thailand") OR EXCLUDE (AFFILCOUNTRY , "Chile") OR EXCLUDE (AFFILCOUNTRY , "Indonesia") OR EXCLUDE (AFFILCOUNTRY , "Colombia") OR EXCLUDE (AFFILCOUNTRY , "Argentina") OR EXCLUDE (AFFILCOUNTRY , "Pakistan") OR EXCLUDE (AFFILCOUNTRY , "Jordan") OR EXCLUDE (AFFILCOUNTRY , "Lebanon") OR EXCLUDE (AFFILCOUNTRY , "Nigeria") OR EXCLUDE (AFFILCOUNTRY , "United Arab Emirates"))

Supplementary table 3. Quality appraisal of included qualitative studies, assessed following the SURE checklist.

Author (Year)	Does the study address a clearly focused question/hypothesis?<br>D1	Is the choice of qualitative methodology appropriate?<br>D2	Is the sampling strategy clearly described and justified?<br>D3	Is the method of data collection well described?<br>D4	Is the relationship between the researcher(s) and participants explored?<br>D5	Are ethical issues explicitly discussed?<br>D6	Is the data analysis/interpretation process described and justified?<br>D7	Are the findings credible?<br>D8	Is any sponsorship/conflict of interest reported?<br>D9	Did the authors identify any limitations?<br>D10
Aumann (2016)[40]	+	+	+	+	–	+	+	+	+	+
Bergqvist (2017)[41]	+	+	?	+	–	+	+	+	+	+
Dalhammar (2023)[30]	+	+	+	+	+	+	+	+	–	+
Dobrina (2016)[36]	+	+	+	+	–	+	+	+	–	+
Dunham (2017)[47]	+	+	?	+	?	+	?	?	–	+
Erol (2018)[48]	+	+	?	+	–	+	+	+	+	+
Håkanson (2015)[42]	+	+	+	+	+	+	+	+	+	+
Hofheinz (2016)[31]	+	+	+	+	?	+	?	?	+	+
IvzoriErel (2022)[37]	+	+	+	+	?	+	+	+	+	+
López-Salas (2024)[38]	+	+	+	+	+	+	+	–	+	+
Laursen (2019)[32]	+	+	?	+	+	+	+	+	+	+
Loughran (2019)[33]	+	+	+	+	+	+	+	+	+	+
Madsen (2019)[34]	+	+	–	+	?	+	+	+	+	+
Maersk (2018)[43]	+	+	+	+	?	+	+	?	–	+
Nysæter (2022)[44]	+	+	+	+	?	+	+	+	+	+
Peoples (2017)[45]	+	+	+	+	–	+	+	+	+	+
Rodríguez-Prat (2022)[35]	+	+	?	+	–	+	+	+	+	+
Rohde (2017)[49]	+	+	+	+	+	+	+	+	+	+
Sonderup Tarp (2024)[46]	+	+	+	+	–	+	+	+	–	+
Stanze (2019)[39]	+	+	+	+	–	+	+	+	+	+

Supplementary table 4. Results of thematic analysis of studies focused on treatment, services and self-management.

**A. PSYCHOLOGICAL FUNCTION (6 themes)**	
**A.1. CANCER-RELATED ANXIETY AND DISTRESS (4 themes)**	
*Personal motives and goals[41]	New value in life; Cancer symptoms as triggers of death anxiety; External motives for treatment.
Being left in distress with unmet needs[42]	"It would be lovely if they wanted to give me a shave. But you can't wait a week because that's too long. I've said that to them every three days. They were going to write everything else down in some book, but they must have forgotten."
Displaying and hiding symbols of identity[43]	"It [home] reflects that I used to be a stickler for order; it is a part of my personality. My home is also an expression of my love of things that are beautiful and aesthetic."
*Existential, social, and psychological symptoms[46]	"But I try to keep my spirits up, because it's no use letting everything be deadly serious. You could easily let yourself fall down that big, black hole, but that wouldn't help a thing."
**A.2. COPING MECHANISMS (1 theme)**	
Self-developed strategies to manage occupations[45]	Dealing with occupational challenges; Being able to take care of one self; Requiring support from others.
**A.4. FEARING AND EXPECTING DEATH (1 theme)**	
*Personal motives and goals[41]	Death as a threat; Cancer symptoms as triggers of death anxiety.
**B. CLINICAL MANAGEMENT (13 themes)**	
**B.1. HEALTH CARE (7 themes)**	
*Experiences with health insurance [40]	Travel costs.
Experiences and preferences during the treatment day [40]	Waiting times.
*Treatment-related experiences and preferences of the patients that influence psychosocial factors[40]	Patients reported many physical side-effects (general sickness, low load capacity, absence of appetite due to the chemotherapy). Problems with changes in their external appearance because of hair loss or skin rashes were also mentioned. These side-effects caused great physical limitations resulting in lower performance levels and flexibility. As a result, patients reported a decrease in sporting and household activities.
The treatment itself [41]	The experience; Stopping treatment is no option; Treatment recovery period.
Being comforted and relieved in bodily care situations[42]	Bodily care was for the most part described as providing general well-being: "I have never been spoiled, so it's [having his body cared for] kind of, it's like they [the nurses] are looking after me"
The assessment of symptoms (questionnaires) [46]	"It was a bit meaningless. I've filled them out and answered to the best of my abilities, and handed them in, and then I haven't heard anything about it … So probably they've been a bit meaningless because they haven't been evaluated afterwards. I assumed they were used as a general evaluation or for statistics."
The treatment of physical symptoms[46]	In general, patients were content with the treatment of physical symptoms. There was a sense of "easy access" to medicine aimed at relieving physical symptoms.

**B.2. INFORMATION & COMMUNICATION (3 themes)**	
*Experiences with physicians [40]	Information about the side-effects of the treatment options; improving information about the changing physicians during treatment.
*Experiences with physicians [40]	Individual arrangements regarding communication methods between the physician and patient.
The decision process[41]	"Actually, you can´t decide yourself, the doctors are best suited for the decision I am not an expert, the doctors have to decide and they do."
**B.3. END-OF-LIFE CARE SETTING (3 themes)**	
Maintaining the privacy of home[43]	"I thought it was strange too, having her messing about with my quilt and pillow. It felt very wrong. I still feel that way."
Managing the home to enable activities[43]	"Usually, I eat at the dining room table. I appreciate anything that can make my life normal. Then I am not sick. Then I don't feel sickly"
Hope and trust to get the care I need to die at home [44]	The patients repeatedly interviewed, continued to express a wish to get End-Of-Life home care and to die at home. Moreover, the hope and trust to get the care they preferred in accordance with their needs were maintained.
**C. SYMPTOMS AND PHYSICAL FUNCTION (4 themes)**	
*Treatment-related experiences and preferences of the patients that influence psychosocial factors [40]	Patients reported many physical side-effects (general sickness, low load capacity, absence of appetite due to the chemotherapy). Problems with changes in their external appearance because of hair loss or skin rashes were also mentioned. These side-effects caused great physical limitations resulting in lower performance levels and flexibility. As a result, patients reported a decrease in sporting and household activities.
Maintaining and losing body capability[42]	"The first time, I tried to wash my hair myself, but I found it a struggle. It took so much effort—I felt I don't have the strength to manage this hair palaver. Anita [first name of nurse] washed me the other day, and I'm fine with that. You get used to it. Strangely enough, you do."
Breaching borders of bodily integrity[42]	"They [nurses] are very cautious and ask me the whole time, "Is that okay, is that okay?" (. . .) It was a lot worse at the start. Now it's kind of nothing really. So you get used to it."
Conditions influencing occupations in everyday life[45]	Continuing everyday life; Leading a quiet life with few occupations; Experiencing loss of occupations.<br>"My life changed from black to white. I used to go to work and was active around the house and the garden. [But now] I´m lucky if I have two-five minutes to do something and then I´m finished; It used to be me who made the breakfast every morning, something I have done since we got married. Now it´s my wife who has to make it"
**D. SOCIAL FUNCTION (1 theme)**	
*Existential, social, and psychological symptoms [46]	"We have chosen to inform the kids every time there's been something to inform about. Had it been better not to say, that now they think it has spread to the liver? Then maybe they wouldn't be scared. I don't know if that has been the right choice."
**E. SPIRITUAL (1 theme)**	
*Existential, social, and psychological symptoms[46]	"No doubt I'm a Christian, and God will get a proper ass-kicking when I see him, because this is completely unfair, I'm so happy with my life"

*Themes categorized into more than one category or subcategory according to the content of the subthemes.

Supplementary table 5. Results of thematic analysis of studies focused on pain.

**B. CLINICAL MANAGEMENT (2 themes)**	
**B.2. INFORMATION & COMMUNICATION (1 theme)**	
Patients' perspectives about nurses' approaches to pain [48]	Perspectives about the nurses' pain assessment.
**B.1. HEALTH CARE (1 theme)**	
Pain management and management strategies [48]	Non-pharmacologic approaches; Pharmacologic approaches.
**A. PSYCHOLOGICAL FUNCTION (5 themes)**	
**A.2. COPING MECHANISMS (1 themes)**	
*Maintaining control and independence [47]	"...I've got a lot of puzzle books in my bedroom... I've got an exercise bike what I bought with rowing things on it..."
**A.1. CANCER-RELATED ANXIETY AND DISTRESS (3 themes)**	
Better to be old than to be dying with cancer [47]	"Well yeah, I've slowed down a lot, I've had to slow down because the body won't take it. It wouldn't take it anyway, not at my age. You think it will, your mind says get on but your body won't take it, so I've had to cut my cloth a little bit and I'm not happy about it."
Loss of identity-adapting and grieving for a former self [47]	"When I'd finished work, before I had my dinner, I'd go out and probably run about four or five miles to have a bit of training."
*Effects of pain on daily life [48]	Powerlessness
**A.3. CONTROL AND DECISION MAKING (1 theme)**	
*Maintaining control and independence[47]	"...I can yes, I can decide for myself, so they're supposed to talk to me first, not to (other family members)."
**C. SYMPTOMS AND PHYSICAL FUNCTION (4 themes)**	
Denial of pain[47]	Pain was denied in several ways. The language used was individual to the person; discomfort, ache and other less commonly used words were used to describe pain.
Dislike of analgesia [47]	All the participants disliked analgesia and they had a complicated relationship with the taking of analgesia.
Pain perception and patient experiences [48]	The meaning of pain; Thoughts about the reason of pain; Past experiences about pain
*Effects of pain on daily life [48]	Fatigue/tiredness; Restrictions

*Themes categorized into more than one category or subcategory according to the content of the subthemes.

Supplementary table 6. Results of thematic analysis of studies focused on spiritual well-being.

**D. SOCIAL FUNCTION (1 theme)**	
Relationships with self and others[49]	Strategies for inner harmony; Sharing feelings with significant others
**E. SPIRITUAL (2 themes)**	
Existential issues[49]	Coping with end-of-life thoughts
Specifically religious and/or spiritual beliefs and practices[49]	Seeking faith as inner support

Supplementary checklist 1

Reporting checklist for systematic review (with or without a meta-analysis).

Title: Needs and health-related quality of life domains relevant to people in Europe with advanced cancer in palliative care: A systematic review of qualitative research

		Reporting Item	Page Number
**Title**			
Title	#1	Identify the report as a systematic review	1
**Abstract**			
Abstract	#2	Report an abstract addressing each item in the PRISMA 2020 for Abstracts checklist	4
**Introduction**			
Background/rationale	#3	Describe the rationale for the review in the context of existing knowledge	6
Objectives	#4	Provide an explicit statement of the objective(s) or question(s) the review addresses	7
**Methods**			
Eligibility criteria	#5	Specify the inclusion and exclusion criteria for the review and how studies were grouped for the syntheses	8
Information sources	#6	Specify all databases, registers, websites, organisations, reference lists, and other sources searched or consulted to identify studies. Specify the date when each source was last searched or consulted	8
Search strategy	#7	Present the full search strategies for all databases, registers, and websites, including any filters and limits used	8
Selection process	#8	Specify the methods used to decide whether a study met the inclusion criteria of the review, including how many reviewers screened each record and each report retrieved, whether they worked independently, and, if applicable, details of automation tools used in the process	9
Data collection process	#9	Specify the methods used to collect data from reports, including how many reviewers collected data from each report, whether they worked independently, any processes for obtaining or confirming data from study investigators, and, if applicable, details of automation tools used in the process	9
Data items	#10a	List and define all outcomes for which data were sought. Specify whether all results that were compatible with each outcome domain in each study were sought (for example, for all measures, time points, analyses), and, if not, the methods used to decide which results to collect	9

Data items	#10b	List and define all other variables for which data were sought (such as participant and intervention characteristics, funding sources). Describe any assumptions made about any missing or unclear information	9
Study risk of bias assessment	#11	Specify the methods used to assess risk of bias in the included studies, including details of the tool(s) used, how many reviewers assessed each study and whether they worked independently, and, if applicable, details of automation tools used in the process	9
Effect measures *(Outcomes)*	#12	Specify for each outcome the effect measure(s) (such as risk ratio, mean difference) used in the synthesis or presentation of results	9
Synthesis methods	#13a	Describe the processes used to decide which studies were eligible for each synthesis (such as tabulating the study intervention characteristics and comparing against the planned groups for each synthesis (item #5))	10
Synthesis methods	#13b	Describe any methods required to prepare the data for presentation or synthesis, such as handling of missing summary statistics or data conversions	NA
Synthesis methods	#13c	Describe any methods used to tabulate or visually display results of individual studies and syntheses	10
Synthesis methods	#13d	Describe any methods used to synthesise results and provide a rationale for the choice(s). If meta-analysis was performed, describe the model(s), method(s) to identify the presence and extent of statistical heterogeneity, and software package(s) used	10
Synthesis methods	#13e	Describe any methods used to explore possible causes of heterogeneity among study results (such as subgroup analysis, meta-regression)	NA
Synthesis methods	#13f	Describe any sensitivity analyses conducted to assess robustness of the synthesised results	10
Reporting bias assessment	#14	Describe any methods used to assess risk of bias due to missing results in a synthesis (arising from reporting biases)	10
Certainty assessment	#15	Describe any methods used to assess certainty (or confidence) in the body of evidence for an outcome	NA
**Results**			
Study selection	#16a	Describe the results of the search and selection process, from the number of records identified in the search to the number of studies included in the review, ideally using a flow diagram (http://www.prisma-statement.org/PRISMAStatement/FlowDiagram)	11
Study selection	#16b	Cite studies that might appear to meet the inclusion criteria, but which were excluded, and explain why they were excluded	NA

Study characteristics	#17	Cite each included study and present its characteristics	11
Risk of bias in studies	#18	Present assessments of risk of bias for each included study	11
Results of individual studies	#19	For all outcomes, present for each study (a) summary statistics for each group (where appropriate) and (b) an effect estimate and its precision (such as confidence/credible interval), ideally using structured tables or plots	11-12
Results of syntheses	#20a	For each synthesis, briefly summarise the characteristics and risk of bias among contributing studies	12-14
Results of syntheses	#20b	Present results of all statistical syntheses conducted. If meta-analysis was done, present for each the summary estimate and its precision (such as confidence/credible interval) and measures of statistical heterogeneity. If comparing groups, describe the direction of the effect	NA
Results of syntheses	#20c	Present results of all investigations of possible causes of heterogeneity among study results	12
Results of syntheses	#20d	Present results of all sensitivity analyses conducted to assess the robustness of the synthesised results	NA
Risk of reporting biases in syntheses	#21	Present assessments of risk of bias due to missing results (arising from reporting biases) for each synthesis assessed	11
Certainty of evidence	#22	Present assessments of certainty (or confidence) in the body of evidence for each outcome assessed	NA
**Discussion**			
Results in context	#23a	Provide a general interpretation of the results in the context of other evidence	14
Limitations of included studies	#23b	Discuss any limitations of the evidence included in the review	16
Limitations of the review methods	#23c	Discuss any limitations of the review processes used	16
Implications	#23d	Discuss implications of the results for practice, policy, and future research	16
**Other information**			
Registration and protocol	#24a	Provide registration information for the review, including register name and registration number, or state that the review was not registered	8
Registration and protocol	#24b	Indicate where the review protocol can be accessed, or state that a protocol was not prepared	8
Registration and protocol	#24c	Describe and explain any amendments to information provided at registration or in the protocol	NA

Support	#25	Describe sources of financial or non-financial support for the review, and the role of the funders or sponsors in the review	23
Competing interests	#26	Declare any competing interests of review authors	23
Availability of data, code, and other materials	#27	Report which of the following are publicly available and where they can be found: template data collection forms; data extracted from included studies; data used for all analyses; analytic code; any other materials used in the review	NA

The PRISMA checklist is distributed under the terms of the Creative Commons Attribution License CC-BY. This checklist can be completed online using https://www.goodreports.org/, a tool made by the EQUATOR Network in collaboration with Penelope.ai
